# The chicken left right organizer has nonmotile cilia which are lost in a stage-dependent manner in the *talpid^3^* ciliopathy

**DOI:** 10.1002/dvg.22775

**Published:** 2014-04-15

**Authors:** Louise A Stephen, Edward J Johnson, Gemma M Davis, Lynn McTeir, Jamie Pinkham, Neema Jaberi, Megan G Davey

**Affiliations:** 1 Division of Developmental Biology, The Roslin Institute and R(D)SVS, University of EdinburghEaster Bush, Midlothian, United Kingdom; 2 Cell and Developmental Biology, College of Life Sciences, University of DundeeDundee, United Kingdom

**Keywords:** TALPID3, KIAA0586, FOXJ1, SHH, PCP, laterality

## Abstract

Motile cilia are an essential component of the mouse, zebrafish, and *Xenopus laevis* Left Right Organizers, generating nodal flow and allowing the reception and transduction of mechanosensory signals. Nonmotile primary cilia are also an important component of the Left Right Organizer's chemosensory mechanism. It has been proposed in the chicken that signaling in Hensen's node, the Left Right Organizer of the chicken, is independent of cilia, based on a lack of evidence of motile cilia or nodal flow. It is speculated that the *talpid^3^* chicken mutant, which has normal left–right patterning despite lacking cilia at many stages of development, is proof of this hypothesis. Here, we examine the evidence for cilia in Hensen's node and find that although cilia are present; they are likely to be immotile and incapable of generating nodal flow. Furthermore, we find that early planar cell polarity patterning and ciliogenesis is normal in early *talpid^3^* chicken embryos. We conclude that patterning and development of the early *talpid^3^* chicken is normal, but not necessarily independent of cilia. Although it appears that Hensen's node does not require motile cilia or the generation of motile flow, there may remain a requirement for cilia in the transduction of SHH signaling.

**Results:**

- *FOXJ1* is expressed at low levels in the chicken node incompatible with motile cilia generation
- Short cilia are present in the mesodermal cells of the chicken node
- *Talpid^3^* chicken embryos have normal VANGL2 localization early in development
- *Talpid^3^* chicken embryos have primary cilia early in development

## INTRODUCTION

*TALPID3* (*KIAA0586*) is an essential gene for vertebrate development. A loss of function mutation in TALPID3 was first mapped in the recessive, embryonic lethal, polydactylous *talpid^3^* chicken breed (Davey *et al*., 2006), and TALPID3 has since been shown to be essential for motile and nonmotile ciliogenesis in the mouse, chicken, and zebrafish (Bangs *et al*., 2011; Ben *et al*., 2011; Stephen *et al*., 2013; Yin *et al*., 2009). A loss of TALPID3 causes a failure of centrosome migration and docking prior to ciliogenesis (Stephen *et al*., 2013; Yin *et al*., 2009). Comparisons of the phenotypes caused by a loss of TALPID3 function in the mouse, zebrafish, and chicken are similar, suggesting an abnormality in Hedgehog signal transduction. All species demonstrate a loss of asymmetry in the developing limb/fin bud preceding the development of unpatterned polydactyly (mouse and chicken) and a loss of *PTCH1* expression in areas expressing Hedgehog ligands. Other phenotypes associated with abnormal Hedgehog signaling include a loss of ventral neuronal patterning in the neural tube of all species, expansion of *Eng2* in the zebrafish somite and misprocessing of Gli2 and Gli3 (Bangs *et al*., 2011; Ben *et al*., 2011; Davey *et al*., 2006). The Hedgehog pathway related phenotypes caused by TALPID3 loss of function are similar to mouse mutants with a loss of primary cilia (e.g., Huangfu and Anderson 2005; reviewed Goetz and Anderson, 2010) as the Hedgehog pathway component proteins, Smo and Gli must be transported into the cilia to activate normal Gli protein modification (e.g., Corbit *et al*., 2005; Haycraft *et al*., 2005; Liem *et al*., 2009; Rohatgi *et al*., 2007). In addition to a loss of Hedgehog pathway function, the *talpid^3^* chicken develops polycystic kidneys and the targeted deletion of TALPID3 in zebrafish causes a dilation of the pronephric duct (Bangs *et al*., 2011; Ben *et al*., 2011). This phenotype is not directly caused by a loss of cilia, but due to a loss of planar cell polarity (PCP) or control of cell cycle associated with abnormal centrosome function, which subsequently contributes to the loss of ciliogenesis (reviewed Hildebrandt *et al*., 2011). All *Talpid3* mutant phenotypes between the three species are consistent with a loss of centrosome or cilia function bar one; while both the zebrafish and the mouse display a loss of cilia on node cells and disrupted left–right asymmetry, the *talpid^3^* chicken heart and gut loop normally, suggesting that left–right asymmetry is normal (Bangs *et al*., 2011).

The developing chicken embryo has been an important model in deducing the role of the node in development, including elucidating the mechanisms patterning vertebrate left–right asymmetry (Levin *et al*., 1995; Männer, 2001; Psychoyos and Stern, 1996; reviewed Davey and Tickle, 2007). It is, therefore, critical to understand the differences between birds and other species if these models are to be used interchangeably, or if insights from one species are to be understood in the context of another. Vertebrate left–right patterning is thought to be established by a number of steps; determining the left–right axis in relation to the anterior:posterior and dorsal:ventral axis or “breaking bilateral symmetry” followed by asymmetrical changes in gene expression and asymmetrical morphogenesis (Nakamura and Hamada, 2012; Vandenberg and Levin, 2013). The mechanisms by which bilateral symmetry is broken are the least understood and have not been clearly determined in zebrafish, chicken, or mammals. It has been proposed that localized protein gradients within the chicken egg (Eyal-Giladi and Fabian, 1980; Sheng, 2014) may establish bilateral differences as early as the first cell cleavage, as is observed in *Xenopus* embryos (Vandenberg and Levin, 2012). This is supported by analysis of avian bilateral gynandromorphs, mixed-sex chimeras which occur due to a failure of polar body extrusion during meiosis. Avian gynandromorphs exhibit distinct male/female trait demarcation along the midline (Aw and Levin, 2008; Vandenberg and Levin, 2013) suggesting that the midline and, therefore, a distinct left–right axis, is established at the first cell division. Analysis of incomplete chicken gynandromorphs, however (Zhao *et al*., 2010), suggests that asymmetry is not established until the eight-cell stage (Ma, 2013). Subsequent to establishing the midline, it has been demonstrated that PCP signaling during the formation of the primitive streak (1–3HH; Hamburger and Hamilton, 1951), establishes the polarization of cells toward the primitive streak and, therefore, confers unique cellular identity in the left and right axis, perturbation of which disrupts subsequent asymmetrical expression of *SHH* (Zhang and Levin, 2009). A role for PCP in patterning the mouse blastoderm prior to ciliogenesis has not been shown, although it has been suggested that many mouse mutants lacking normal left–right axis specification, may do so because they lack normal PCP signaling. However, as ciliogenesis is also effected in these mutants the exact role for PCP patterning in the mouse is currently unclear (Vandenberg and Levin, 2013). Subsequently, mice, zebrafish, and *Xenopus* are all thought to require the action of motile nodal cilia in breaking symmetry (Nakamura and Hamada, 2012), whereas in the chicken, symmetry is thought to be broken at Hensen's node by cell movements (Gros *et al*., 2009) following PCP signaling, controlled by the asymmetrical activity of H+/K+ ATPase (Levin *et al*., 2002). It has been suggested that cilia are unlikely to play a role in breaking symmetry in the chick node (Levin and Palmer, 2007).

The divergent lack of a requirement for cilia, the type and even existence of cilia in Hensen's node is controversial. Monocilia have been observed in a SEM study of Hensen's node, albeit rarely (Männer, 2001). Although it has been reported that the motile cilia component *DNAH9/LRDR1* is expressed in Hensen's node and that morphologically these cilia appear unlike motile cilia found on the mouse node (Essner *et al*., 2002), a subsequent report has suggested that these cilia were endodermally localized rather than on the mesodermal cells of the node (Gros *et al*., 2009). Furthermore, the mesoderm of the chicken node is bound by epiblast/ectoderm dorsally and endoderm ventrally so does not present an exposed surface within a pit in which nodal flow might be generated by motile cilia (Männer, 2001). Thus, it is unclear whether there are any cilia on chicken mesodermal node cells, of either motile or nonmotile type. It has been proposed that the *talpid^3^* chicken phenotype is, therefore, evidence that cilia and, therefore, nodal flow are not components in determining left–right asymmetry in chickens (Gros *et al*., 2009). In contrast to the chicken, the mouse node is an exposed pit on the ventral side of the embryo, from which precisely angled motile cilia project to create a directed flow of extraembryonic fluid toward the left of the embryo (Nonaka *et al*., 1998; Nonaka *et al*., 2005). This flow is detected by immotile mechanosensory cilia, found on the crown cells lining the outer edge of the node (McGrath *et al*., 2003; Yoshiba *et al*., 2012; reviewed Nakamura and Hamada, 2012). Generation and detection of nodal flow in the mouse is vital in inducing sided expression of laterality markers, including *Nodal*, *Lefty*, and *Pitx2* (Nonaka *et al*., 1998; Nonaka *et al*., 2002).

The maintenance of bilateral asymmetry throughout development requires a highly conserved cascade of gene expression, which firmly establishes left versus right morphology, although the details of regulation differ between species. *SHH* is expressed at Hensen's node in response to cues from adjacent tissue (Levin *et al*., 1995; Pagán-Westphal and Tabin, 1998; Yuan and Schoenwolf, 1998). Like the mouse, *SHH* is initially expressed symmetrically about the chicken node at 4^−^HH but becomes asymmetric by 4^+^HH through asymmetric cell movements at the node (Gros *et al*., 2009; Levin *et al*., 1997). Left sided *SHH* expression subsequently induces *NODAL* expression in the left lateral plate mesoderm (Gros *et al*., 2009; Levin *et al*., 1995; Pagán-Westphal and Tabin, 1998) which induces downstream expression of *LEFTY2* (Meno *et al*., 1997) and *PITX2* (Yu *et al*., 2001). The maintenance of this molecular cascade is reliant not only on upstream expression of laterality markers but also on cell–cell interactions and integrity of tissue and cell junctions in the early embryo (Levin and Mercola, 1999; Yuan and Schoenwolf, 1998).

In the mouse, *Shh* is expressed through the midline, inducing expression of *Lefty1* in the floorplate, where it prevents expression of left-sided markers on the right-hand side (Chiang *et al*., 1996; Lee and Anderson, 2008; Meyers and Martin, 1999; Tsukui *et al*., 1999). Expression of *Nodal* is first seen throughout the epiblast in the preimplantation embryo (Brennan *et al*., 2001), before being induced in the perinodal cells of the mouse at the early headfold stage, by a node-specific enhancer, proposed to be induced by *Notch* signaling ligand DII1 (Conlon *et al*., 1994; Krebs *et al*., 2003; Raya *et al*., 2003). Perinodal expression of *Nodal* in turn induces *Nodal* expression in the left lateral plate mesoderm (Norris and Robertson, 1999; Saijoh *et al*., 2003) where it acts first to maintain its own expression, but also to induce expression of *Pitx2* and *Lefty2* (Logan *et al*., 1998; Meno *et al*., 2001). Expression of *Lefty2* is transient in the lateral plate mesoderm and produces a negative feedback loop with *Nodal*, preventing overexpression in the lateral plate mesoderm (Chen and Shen, 2004), while *Pitx2* expression is maintained in the developing asymmetric organs including the heart as it undergoes looping (Campione *et al*., 1999; Logan *et al*., 1998).

Whether or not cilia play a role in breaking symmetry in the node, the canonical transduction of Hedgehog signaling in vertebrates is dependent on primary cilia (Goetz and Anderson, 2010). It would, therefore, be an exceptional situation for cilia not to be an essential component in transducing the SHH signal in the node of the chicken. The *talpid^3^* chicken which maintains normal left–right patterning despite exhibiting ciliogenesis and PCP defects and abnormal Hedgehog signal transduction is therefore an outstanding enigma in this field. To determine how current models integrate with the observed patterning in *talpid^3^*, we have undertaken a study of PCP patterning, asymmetrical gene expression analysis, and Hedgehog pathway transduction in *talpid^3^* embryos.

## RESULTS

### *Talpid^3^* Chicken Embryos have Normal Asymmetrical Organ Patterning, whereas *Talpid3^−/−^* Mice do not

**Figure 1:**
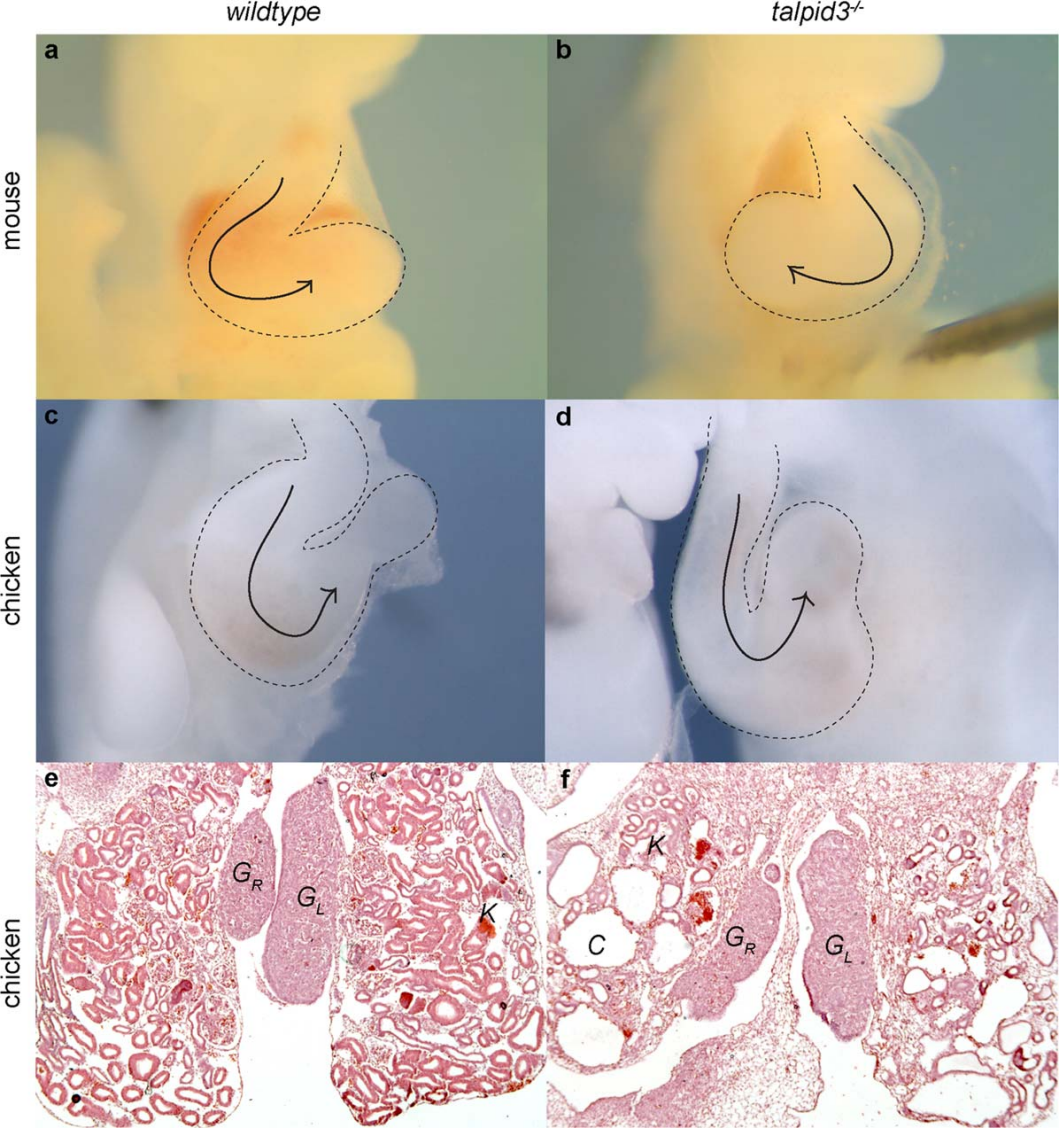
Organ asymmetry is abnormal in *Talpid3^−^^/^^−^* mouse, but normal in *talpid^3^* chicken embryos. *Wildtype* mouse and chicken embryos exhibit dextral cardiac looping (a, c). This is reversed in the *Talpid3^−^^/^^−^* mouse (b) but not in the *talpid^3^* chicken (d). Regression of the right gonad occurs in both the *wildtype* and *talpid^3^* chicken by E10 (e, f). Abbreviations: G_R,_ Right gonad; G_L_, Left gonad; C, Kidney cyst; K, Kidney. a–d magnification comparable, e, f magnification comparable.

In the early mouse and chicken, heart morphology is a key indication of left–right asymmetry. The heart begins as a linear heart tube at around E8.5 in the mouse (reviewed Harvey, 2002) and 12HH in the chicken (Männer, 2000). The tube then begins to loop dextrally to bring the primitive chambers of the heart into the correct configuration. We have previously reported that the *Talpid3^−^^/^^−^* mouse exhibits randomization of laterality in abnormal cardiac looping (Bangs *et al*., 2011), whereas the *talpid^3^* chicken heart has normal heart looping, liver lobe specification and stomach turning, although the sample numbers were low (*n* = 2 for liver and stomach patterning). We confirm here that compared to *wildtype* mice (Fig. 1a) heart looping in the *Talpid3^−^^/^^−^* mouse at E9.5 stage is randomized (Fig. 1b). .08% of *Talpid3^−^^/^^−^* mouse embryos exhibited normal dextral looping (*n* = 19), 19.23% exhibited reversed, leftwards looping (*n* = 5) and 7.69% failed to undergo looping (*n* = 2). At 12HH in *wildtype* and *talpid^3^* chicken embryos, however, heart looping is normal (Fig. 1c,d, *n* > 100).

We further examined *talpid^3^* chicken embryos to determine viscera patterning using liver lobe size (right is larger than left), portal vein location (right lobe), side of stomach location (left), and stomach turning (left) between Day 10 and Day 14 of incubation. Laterality was found to be normal in 100% of *talpid^3^* embryos studied (*n* = 8). In the female chicken, gonad development is dependent on the same molecular cues of laterality (Guioli and Lovell-Badge, 2007; Guioli *et al*., in press) and at E10 the right gonad is smaller than the left, having regressed (review Smith and Sinclair, 2004). At E10, both *wildtype* and *talpid^3^* female embryos exhibit regression of the right gonad (*talpid^3^ n* = 8 as demonstrated through dissection, histology (G_R;_ Fig. 1e,f), and *PITX2* expression (*talpid^3^ n* = 3; not shown).

### Chicken Embryos Exhibit Primary Cilia on the Node Mesoderm

**Figure 2:**
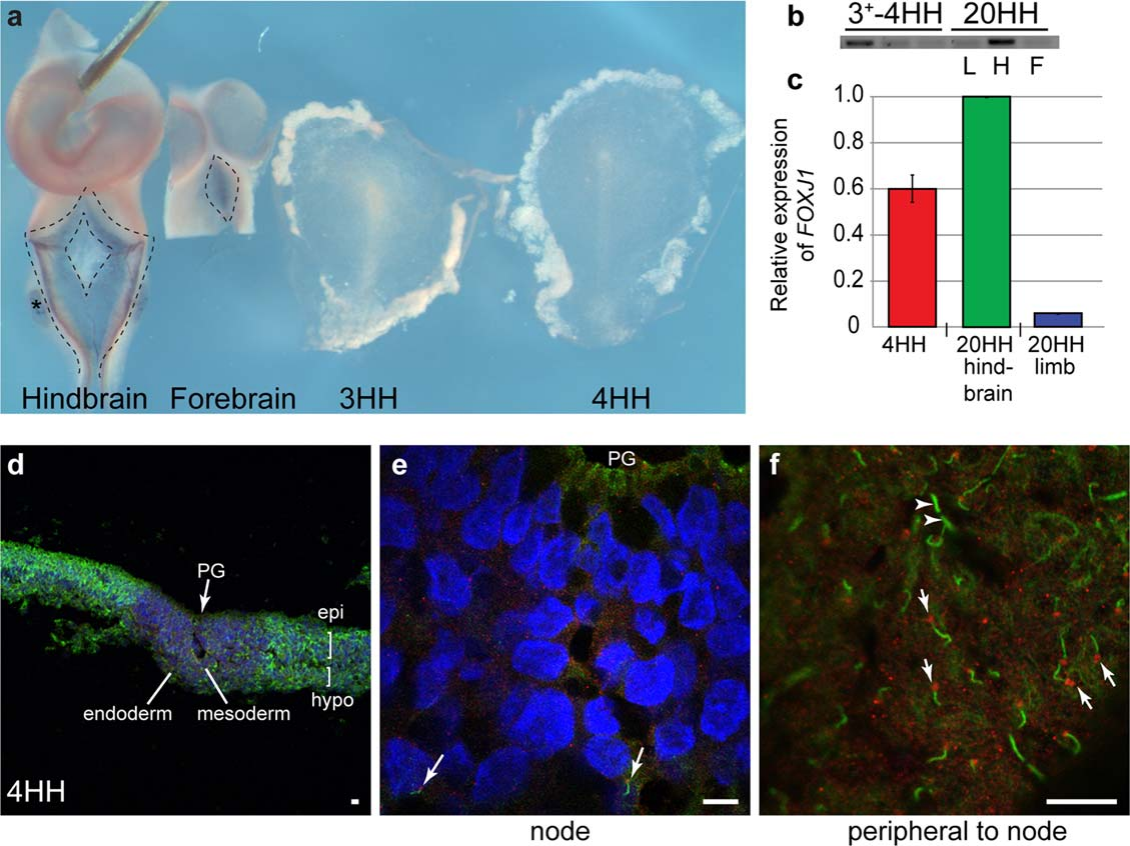
*FOXJ1* expression is not sufficient to induce motile cilia formation in the chicken node. (a) *FOXJ1* expression in the future choroid plexus of hindbrain, forebrain (outlined, arrows), and otic vesicle (asterisk) of stage 20HH embryos which precedes motile ciliogenesis, compared with stage 3HH and 4HH embryos, where no *FOXJ1* can be observed (all embryos photographed underwent RNA in situ hybridization in the same tube). Red arrow indicates node, green arrow indicates primitive streak. (b) Expression of *FOXJ1* by RT-PCR does show expression in stage 3HH and 4HH embryos compared to stage 20HH hindbrain (H) and forebrain (F) [negative control is stage 20HH limb bud (L)]. (c) qPCR, shows that *FOXJ1* at 4HH is approximately 60% of that of the hindbrain (*P* = 0.000245). (d–f) Stage 4HH embryos underwent immunocytochemistry for acetylated tubulin (ciliary axoneme, green) and γ tubulin (basal body, red) to identify cilia (white arrows). Short cilia were identified in the mesoderm of the node (arrows e), while longer cilia were identified in greater abundance, in the epiblast (epi) and hypoblast (hypo) peripheral to the node (arrows f, arrowheads indicate telophase bridges which look similar to cilia but are not associated with a basal body). PG-Primitive groove; d-magnification 10×; e-magnification 50×; f-magnification 100×.

The presence or absence of cilia on the chicken node is controversial in the literature and difficult to ascertain. Initially, we undertook gene expression analysis of *FOXJ1*, a master regulator of motile cilia, as it has been reported that the motile cilia gene *DNAH9*/*LRDR1*, which is normally controlled by *FOXJ1*, is expressed in Hensen's node (Essner *et al*., 2002). Compared to expression in the developing choroid plexus, a tissue which exhibits multiciliated motile cilia (Stephen *et al*., 2013), we were unable to detect *FOXJ1* by wholemount RNA in situ hybridization in the chicken node (Fig. 2a). However RT-PCR from RNA isolated from the primitive streak and tissue immediately lateral to the primitive streak showed expression of *FOXJ1* compared to a negative control of stage 20HH limb bud tissue (Fig. 2b) although qPCR confirmed that this expression was low compared with the choroid plexus (Fig. 2c). This suggests that Hensen's node may express *FOXJ1* and have the potential to develop motile cilia as in the Left Right Organizers of other species. To confirm the presence/absence and type of cilia at Hensen's node, we then undertook immunohistochemistry with acetylated α tubulin (ciliary axoneme) and γ tubulin (basal body at base of cilia) on sectioned 4HH primitive streaks (*n* = 10) to observe cilia of any type (Fig. 2d,e). We observed short, infrequent cilia on nodal mesoderm cells ingressing through the primitive groove (arrows, Fig. 2e). In comparison, lateral to the node, long cilia were present in a high concentration in embryonic epiblast and hypoblast cells (arrows denote cilia, Fig. 2f). All 10 of the embryos analyzed displayed the same distribution of cilia. Therefore, we can confirm that the chicken node does not have motile cilia but does have short cilia, which are likely to be immotile. Lateral to the node, cells have long cilia, which may be the source of *FOXJ1* expression and may, therefore, be motile.

### *Talpid^3^* Chickens Exhibit Cilia before 8HH

**Figure 3:**
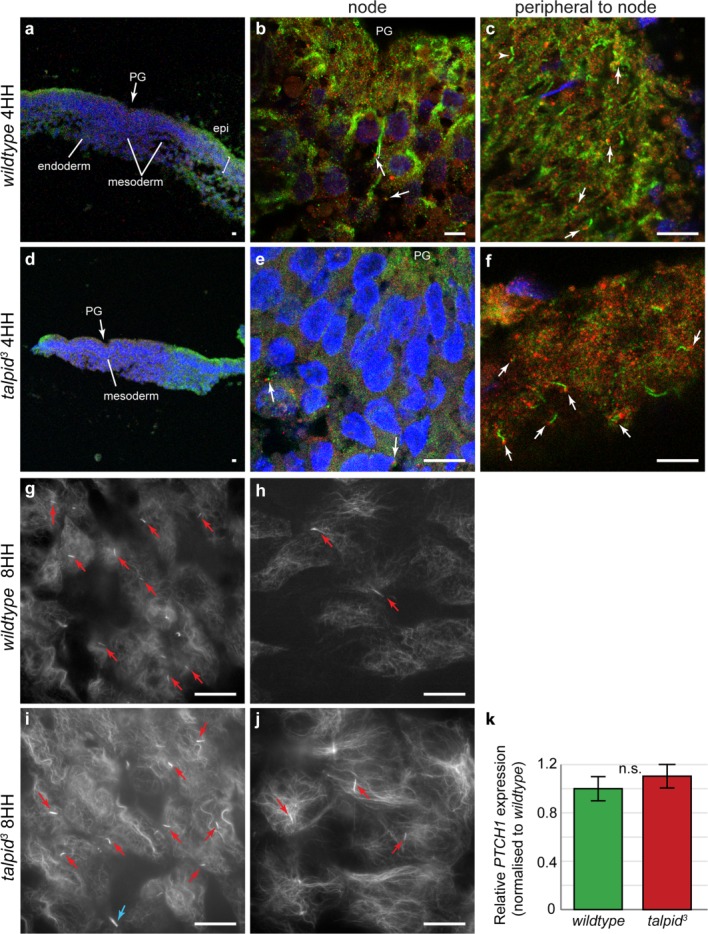
Cilia are present and functional in the node of 4HH-8HH *talpid^3^* chickens. (a–f). Stage 4HH *wildtype* and *talpid^3^* embryos underwent section immunocytochemistry for acetylated tubulin (ciliary axoneme, green) and γ tubulin (basal body, red) to identify cilia (white arrows). Short cilia were identified in the mesoderm of the nodes of both genotypes (b, e, arrows), while longer cilia were identified in greater abundance, in the epiblast (epi) and hypoblast (hypo) peripheral to the node (c, f, arrows. Arrowheads indicate telophase bridges which look similar to cilia but are not associated with a basal body). (g–j). Immunocytochemistry was carried out on 8HH embryos for acetylated tubulin to identify cilia (red arrows; blue arrow denotes telophase bridge). Cilia were identified in both nodal (g, i) and nonnodal tissues (h, j) in both *wildtype* (g, h) and *talpid^3^* (i, j) embryos at 8HH. (k) qPCR show no significant difference between expression of *PTCH1* in *wildtype* and *talpid^3^* embryos (*P* = 0.84). PG-Primitive groove. (a–f) Magnifications comparable: a, d-magnification 10×, b-magnification 50×, c, e, f-magnification 100×. Magnification of g–j the same (not comparable to a–f).

We have previously reported that *talpid^3^* chicken embryos lack cilia at stage 20HH (Yin *et al*., 2009) and have also found they lack cilia at 17HH (Davey *et al*., unpublished). We undertook immunohistochemistry on two *wildtype* and two *talpid^3^* embryos at 4HH (Fig. 3a–f). In agreement with the previous findings, we observed short cilia on the mesoderm under the primitive groove (Fig. 3b) and long cilia lateral to the primitive groove in the epiblast/hypoblast tissue in *wildtype* embryos (Fig. 3c). Surprisingly, we also found cilia on both mesodermal nodal cells (short and infrequent) and in the epiblast/hypoblast (long) in the *talpid^3^* embryos (Fig. 3e,f). To determine if the cilia we observed were transitory, we undertook wholemount immunohistochemistry with antibodies against acetylated α tubulin on wholemount embryos between 6 and 8HH. *Talpid^3^* genotype (*n* = 2) could not be distinguished from *wildtype* (*n* = 6) on the basis of presence, absence or frequency of cilia, demonstrating that *talpid^3^* embryos exhibit cilia until at least 8HH of development. Short primary cilia (red arrows, Fig. 3g,i) were observed in and around the node while longer primary cilia were seen throughout embryonic tissues (red arrows, Fig. 3h,j). Blue arrows denote telophase bridges, which can look similar to cilia but are not associated with a basal body (Fig. 3i).

Hedgehog signaling has been shown to be abnormal due to a loss of primary cilia in stage 20HH *talpid^3^* embryos. We, therefore, undertook qPCR for *PTCH1*, which is expressed in response to normal Hedgehog signal. At stage 3–4HH there was no significant difference (*P* = 0.84) between *PTCH1* expression in *talpid^3^* embryos (*n* = 3) compared to *wildtype* embryos (*n* = 9), suggesting transduction of Hedgehog signaling via the observed primary cilia, is normal at stage 3-4HH in *talpid^3^* embryos (Fig. 3k).

### VANGL2 Localization in Normal in *talpid^3^* Embryos

**Figure 4:**
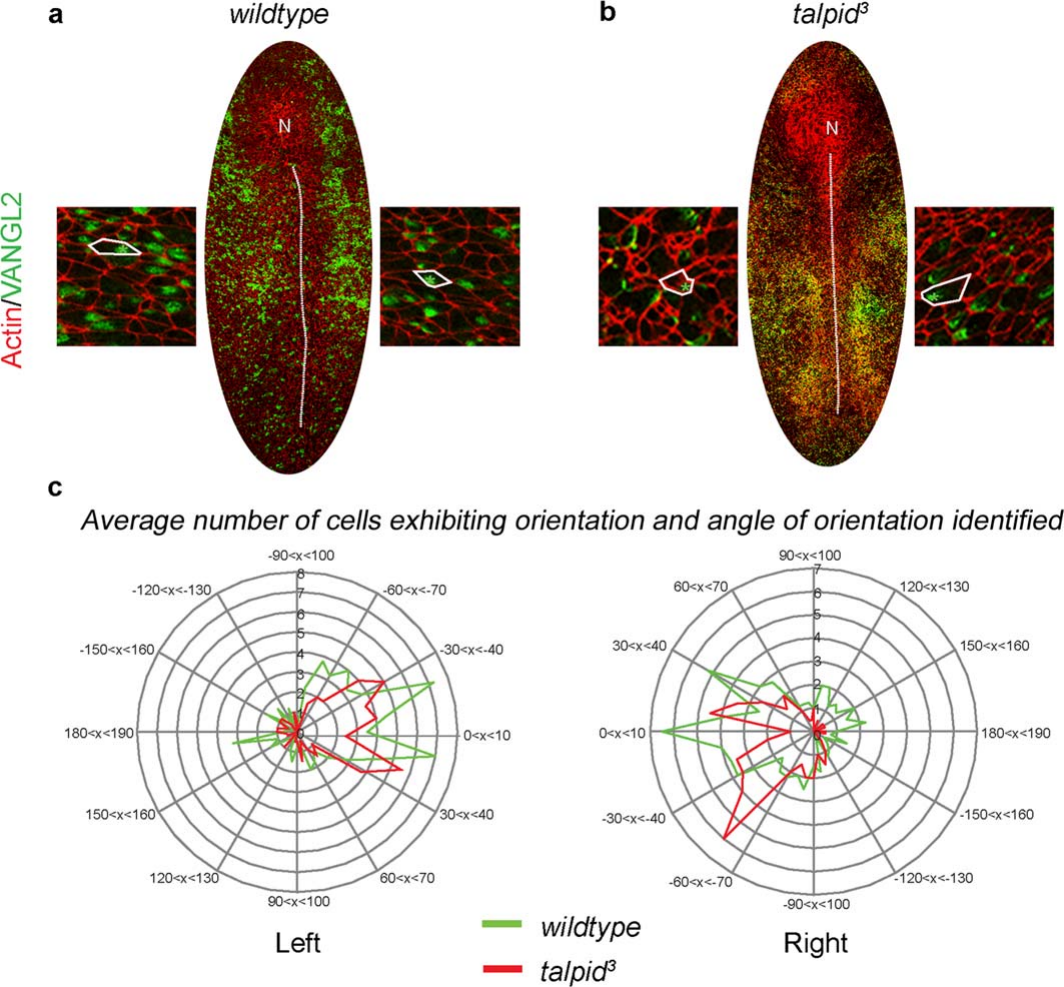
The core PCP component VANGL2 is normally localized in *talpid^3^* chickens at 4HH. (a, b) examples of wholemount labeled 4HH embryos, green = VANGL2 localization, red = actin, outlining the edges within the epiblast. Insets show magnification of cells lateral to the primitive streak and examples of cells outlined in white with a green asterisk highlighting VANGL2 localization. In *wildtype* embryos (a) and *talpid^3^* embryos (b), VANGL2 protein within the cell was orientated toward the primitive streak in 72.4% of embryos (green, c), while 83.58% of *talpid^3^* embryos were orientated toward the primitive streak (red, c). Abbreviations: N, Hensen's Node; dotted line—primitive streak.

Localization of the core PCP pathway component VANGL2 has been shown to be an early step in left–right specification in the chicken embryo (Zhang and Levin, 2009). Furthermore, *talpid^3^* embryos show evidence of PCP defects including a loss of normal centrosome migration and polycystic kidneys (Stephen *et al*., 2013; Yin *et al*., 2009). We, therefore, examined the localization of one of the core components of the PCP pathway, VANGL2 in four *wildtype* embryos and two *talpid^3^* chicken embryos between 4HH (Fig. 4a,b, side panels show examples of cellular localization of VANGL2 from either side of the primitive streak). Analysis of the orientation of VANGL2 at 4HH, with respect to the primitive streak, revealed that a distinct polarity toward the primitive streak was exhibited in 72.4% of *wildtype* cells and 83.58% of *talpid^3^* cells. A chi squared test applied to this data confirmed significant polarization of cells in both *wildtype* (Left side;
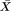
= 42°, *P* < 0.01. Right side;
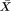
= 79.8°, *P* < 0.001) and *talpid^3^* embryos (Left side;
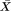
= 60.5°, *P* < 0.001. Right side;
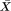
= 54.9°, *P* < 0.001 right) when compared to an expected random distribution, when angles between −90 and 90° indicate orientation toward the primitive streak, positive orientations are directed toward Hensen's node while negative are orientated away from the Hensen's node. A kurtosis (*k*) analysis applied to the data determined *k* values of 1.54/2.71 (left/right) in *wildtype* and 0.71/1.53 (left/right) *talpid^3^* suggesting a strong polarization of the data toward the mean in both phenotypes. A D'Agostino and Pearson omnibus normality test showed genotypes did not statistically differ from one another, or a normal distribution (Left side; *wildtype* = 0.0611, *talpid^3^* = 0.1566. Right side; *wildtype* = 0.4751, *talpid^3^* = 0.1676). Likewise an unpaired, two tailed *t*-test showed no variation in distribution between *talpid^3^*and *wildtype* (left side *P* = 0.2404 and right side *P* = 0.1571). Thus VANGL2 localization is polarized in the cell with respect toward the primitive streak, and that this polarization is not affected by the *talpid^3^* mutation.

Thus, three functions of the TALPID3 protein, ciliogenesis, transduction of Hedgehog signaling, and PCP patterning which have been found to be abnormal in older *talpid^3^* embryos were normal at stage 4HH. We did observe a certain level of actin disorganization in the organization of actin in *talpid^3^* embryos at stage 4HH which has previously been described in older *talpid^3^*chicken embryos (Yin *et al*., 2009), suggesting that not all TALPID3 dependent function may be normal.

### Symmetry is Broken Normally in the *talpid^3^* Chicken

**Figure 5:**
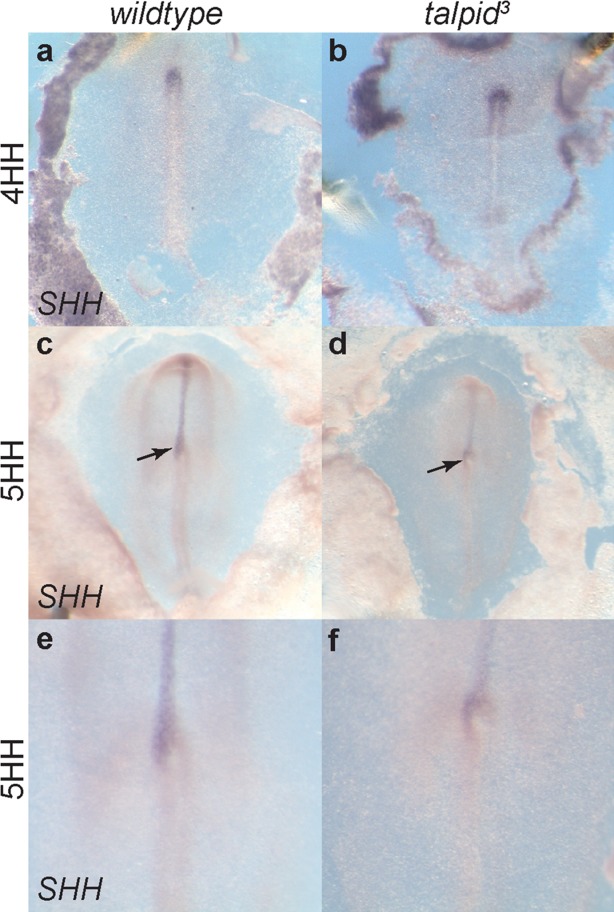
*SHH* expression is normal in the *talpid^3^* chicken at 5HH. SHH is expressed bilaterally at the node, through the rostral notochord at 3–4HH (a, b) but is lateralized, becoming expressed on the left side of the node from 5HH (arrows, c–f) in both *wildtype* (c, e) and *talpid^3^* (d, f) embryos. Magnification a–d comparable (1.6×), e–f (4×).

Having identified normal cell polarization in the epiblast of 4HH chickens, we examined *SHH* expression to observe if symmetry is broken normally in the *talpid^3^* chicken. Expression of *SHH* in *wildtype* and *talpid^3^* embryos is seen throughout the rostral notochord in a bilaterally symmetrical manner between 3 and 4HH (Fig. 5a,b). From 4^+^HH to 8HH *SHH* expression became normally lateralized to the left-hand side of Hensen's node in *wildtype* and *talpid^3^* embryos (arrows, Fig. 5c–f). In summary, in three in situ hybridization experiments encompassing 78 embryos of mixed genotypes, we found no abnormal expression of *SHH* as expression was either found bilaterally 3-4HH or on the left side of the node (Supporting Information Table S1.)

### Lateralized Gene Expression is Normal in *talpid^3^* Chicken Embryos but not *Talpid3^−/−^* Mouse Embryos

**Figure 6:**
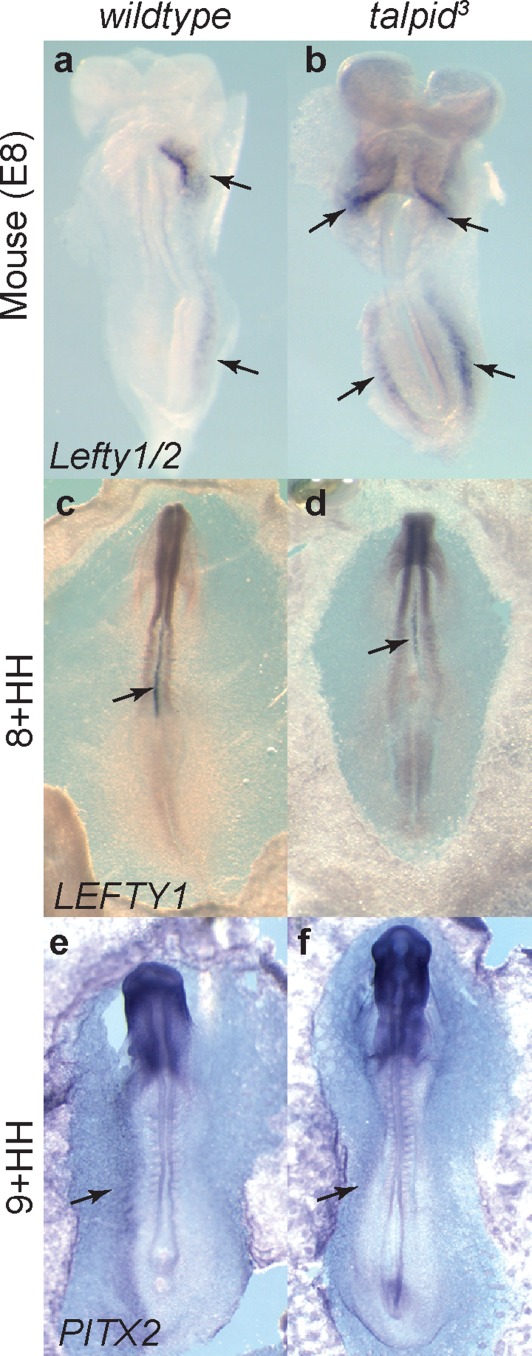
Laterality markers are unperturbed in the *talpid^3^* chicken. In situ hybridization in mouse embryos demonstrated expression of laterality markers *Lefty1/2* in *wildtype* mouse embryos (a), where *Lefty2* is expressed the left lateral plate mesoderm and cardiac folds (arrows) and in the *Talpid3^−^^/^^−^* mouse which exhibits expression bilaterally (arrows, b). *LEFTY1* in *wildtype* chicken embryos at 8HH (c) and *talpid^3^* chicken embryos (d) show the same expression throughout the notochord. *PITX2* is expressed in the left lateral plate mesoderm (arrow e, f) in both the *wildtype* (e) and *talpid^3^* chicken in both *wildtype* and *talpid^3^* chickens.

Having identified normal expression of *SHH* and the presence of cilia in the early *talpid^3^* embryo, we examined expression of downstream laterality markers in *wildtype* and *talpid^3^* embryos. Other areas of Hedgehog signaling have been shown to be abnormal in the *talpid^3^* embryo (Davey *et al*. 2006; Lewis *et al*. 1999), therefore, although *SHH* expression itself is normal, we might expect gene expression downstream of SHH signaling to be perturbed in the *talpid^3^* chicken. *LEFTY1* expression was studied in 83 embryos of mixed genotypes between 3HH-12HH, of which 10 were subsequently confirmed as *talpid^3^* embryos. *LEFTY1* expression was observed from 4HH in the node of both *wildtype* and *talpid^3^* embryos and in the developing primitive streak until 6–7HH, when expression was briefly restricted to the left of the node (not shown). From 8HH expression of *LEFTY1* was found throughout the notochord (arrow, Fig. 6c,d), thus expression of *LEFTY1* was not perturbed in the *talpid^3^* chicken. *Lefty1/2* was also studied in the *Talpid3^−^^/^^−^* mouse. Although expression was found in the left lateral plate mesoderm and cardiac folds of the *wildtype* mouse (arrows Fig. 6a), *Lefty1/2* was expressed in both the left and right lateral plate mesoderm and cardiac folds of the *talpid^3^* mouse (arrows, Fig. 6b, *n* = 1). We also examined *PITX2* expression in 34 embryos between 8HH-11HH over three experiments. *PITX2* expression was consistently identified in the left lateral plate mesoderm in both *wildtype* and *talpid^3^* embryos (Fig. 6e,f) and was, therefore, normal. A breakdown of the numbers of embryos examined for *SHH* and *LEFTY1* with an analysis of genotype and expression pattern can be found in Supporting Information Figure S1.

## DISCUSSION

The role of cilia in the specification of laterality in the chicken is one of the great enigmas of the field. Cilia play a dual role in the mouse, acting both to produce nodal flow and to detect changes caused by that nodal flow, requiring a combination of both long, motile cilia, and shorter sensory cilia (McGrath *et al*., 2003). In contrast, we have found that the chicken has only the shorter cilia on the mesodermal cells of the node. The morphology of these cilia combined with a low *FOXJ1* expression in chicken node cells suggests these cilia are likely to be nonmotile and unable to generate a nodal flow and could still be required for the transduction of Hedgehog signaling at the node. The similarities to the situation in the early pig embryo, where short cilia but not long motile cilia are present, despite low expression of *Foxj1* (Gros *et al*., 2009) suggests that this is unlikely to be an avian specific patterning mechanism and that signaling through short nonmotile cilia combined with cell movements at the node could be used by many vertebrates.

What is perhaps more surprising in this study is the presence of cilia in *talpid^3^* chicken embryos. Previous studies in the *talpid^3^* mutant chicken have identified a complete loss of both motile and primary cilia after 3 days of development (17HH; Stephen *et al*., 2013; Yin *et al*., 2009). In a mouse or zebrafish heterotaxy model in which *situs* is abnormal due to a loss of motile cilia, we might expect to see 33% of embryos with *situs inversus*, and 33% with randomized viscera, while in the classic heterotaxy model, the *iv* mouse, rates of viscera reversal are even higher, with 50% demonstrating reversal, and 40% exhibiting randomization (Layton *et al*., 1993). If heterotaxy were present in the *talpid^3^* chicken model, we would expect a potential of five abnormal embryos in our sample of eight, with at least two exhibiting abnormal liver positioning. As *talpid^3^* mutants do not exhibit any laterality defects; this, in combination with the lack of motile cilia in chicken, has lead other groups to hypothesize that the chicken does not require cilia to initiate bilateral asymmetry (Gros *et al*., 2009). This hypothesis has been supported by the generation of the *Talpid3^-/-^* mouse, which in many ways phenocopies the chicken mutant; exhibiting a loss of cilia due to disrupted centrosomal migration and developing aberrant *Shh* signaling, polydactyly, and loss of facial midline structures (Bangs *et al*., 2011; Davey *et al*., 2006; Hinchliffe and Ede, 1967; Stephen *et al*., 2013; Yin *et al*., 2009). However, unlike the *talpid^3^* chicken, the *Talpid3^−^^/^^−^* mouse exhibits randomization of lateralized structures, suggesting that while the mouse and chicken TALPID3 proteins appear to have the same function, loss of cilia causes laterality defects in the mouse, but not in the chicken (Bangs *et al*., 2011). Similarly, expression of laterality markers; *Nodal* (Bangs *et al*., 2011) and *Lefty1/2* become randomized in the *Talpid3^−^^/^^−^* mouse, while early expression of *SHH*, *LEFTY1*, and *PITX2* is normal in the *talpid^3^* chicken. The identification of cilia in the early *talpid^3^* chicken offers an alternate explanation for the role of the cilia in chicken laterality and demonstrates that cilia in *talpid^3^* chicken embryos at this stage are functional, as *PTCH1* expression is unperturbed and, therefore, receptive to signaling via the cilia in the early embryo.

We suggest that the presence of cilia in the early *talpid^3^* chicken may be due to maternal protein. Of particular interest in this field is the MZ*talpid3* zebrafish mutant. Maternally derived *talpid^3^* mRNA has been identified in the zebrafish *talpid3* model as late as 2 days post fertilization (dpf; Ben *et al*., 2011) and cilia have been found to persist in fish homozygous for the truncated TALPID3 protein, until 4 dpf. Interestingly, these fish exhibit only a very mild, late onset ciliopathy phenotype with no laterality abnormalities. In zebrafish that were also null for maternal *talpid3*, cilia failed to form, and embryos exhibited a more severe ciliopathy phenotype, including randomization of lateralized organs (Ben *et al*., 2011). Human TALPID3 also exhibits an expression profile of a maternal effect protein in human embryos, being most highly expressed in the oocyte, showing gradual reduction between the two-cell and six-cell stage, before an increase again at the eight-cell stage, when zygotic expression is initiated (Human Embryo Resource, 2013; Vassena *et al*., 2011). By RT-PCR (data not shown), we have shown that TALPID3 is expressed at stage 4HH (14/14 embryos). Although we can detect nonsense mediated decay in sequence traces of the RT-PCR product in heterozygous embryos (5/5 embryos), we have not been able to detect a maternal allele in homozygotes through this method. To confirm this hypothesis, we have attempted to identify *wildtype* protein in *talpid^3^* embryos between stage 3–4HH, however, we have been unable to detect TALPID3 protein in either *wildtype* or *talpid^3^* embryos due to its low abundance and/or the small size of the tissues. An alternative hypothesis is that TALPID3 may not be required during early chicken embryonic development though this is unlikely as it is required in both early mouse and zebrafish development and has been shown to be expressed in early human development (Human Embryo Resource, 2013). In addition, TALPID3 has no homologues to compensate for a loss of expression. If a similar maternal effect was present in the chicken embryo, this would explain the presence of cilia, and a subsequent lack of laterality phenotypes in the *talpid^3^* chicken, although it would be surprising that a maternal protein was still active at 8HH/32 h of development (and possibly beyond). There is evidence that functional cilia may be present until 17HH, as we have observed evidence of floorplate induction which requires functional Hedgehog signaling from the notochord (Lewis *et al*., 1999) and weak but significant posterior *PTCH1* expression in the early developing wing bud (Davey, unpublished). Until recently, technology has left the study of maternal effect in chickens far behind that of the zebrafish community. However, the development of a chicken primordial germ cell line (Macdonald *et al*., 2012; van de Lavoir *et al*., 2006) has allowed us to derive *talpid^3^* chicken primordial germ cells and we hope in the future to use this to produce a germ-line replacement *talpid^3^* null to determine whether cilia truly play a role in laterality of the chicken. It will also be interesting to observe left–right patterning defects in other chicken ciliopathies such as *talpid^2^* (Brugmann *et al*., 2010), which might not have a maternal component.

## CONCLUDING REMARKS

Left–right asymmetry in the chicken continues to be an enigma. However, work in the *talpid^3^* chicken suggests that cilia may well have a role to play here as they do in the rest of the vertebrate world. We propose that early presence of *wildtype* TALPID3 protein in the *talpid^3^* chicken underlies normal laterality by permitting the formation of cilia in the early stages of development. This in turn allows the normal transduction of *Shh* protein which initiates normal expression of laterality markers and, therefore, normal asymmetric morphogenesis.

## METHODS

### Chick Embryo Incubation and Dissection

Eggs from *talpid^3^* flock (MG Davey; The Roslin Institute) were incubated at 38°C for 3–12 days, staged as per (Hamburger and Hamilton, 1951). Embryos between 4HH and day 14 were dissected into cold phosphate buffered saline (PBS) and immediately fixed in 4% paraformaldehyde (PFA). Sacrifice of chicken embryos by a schedule one method is not a regulated procedure under UK Home Office rules. The *talpid^3^* flocks were maintained at the Roslin Institute, Edinburgh under a Home Office license and after ethical review. The justification for their maintenance is primarily that they are used as animal models for human conditions.

### Mouse Dissection

Timed matings were established between *Talpid3^+/^^−^* mice (Bangs *et al*., 2011) and mating confirmed by vaginal plug; females sacrificed at Day 8 of pregnancy. Embryos at Embryonic Day 8 (E8) were dissected in cold PBS and fixed immediately in 4% PFA before dehydration through a methanol gradient. The *Talpid3^+/^^−^* mice are maintained on The Roslin Institute breeding and production license 60/4518

### Histology

Chicken embryos at E10 were processed and embedded in paraffin wax, sectioned and stained with haematoxylin and eosin as per (Yin *et al*., 2009).

### Immunofluorescence

For section immunocytochemistry chicken embryos were dissected into PBS, fixed, sectioned, and stained as per (Davey *et al*., 2006). Antibodies used—acetylated α tubulin (Sigma-Aldrich T7451), γ tubulin (Sigma-Aldrich T5192), anti-mouse (Life Technologies A11017), and anti-rabbit (Life Technologies A21207). Phalloidin (Life Technologies A22284). VANGL2 localization was studied in 4HH-8HH wholemount embryos using VANGL2 antibody (Santa Cruz SC-46560) and phalloidin (Life Technologies A22284). Immunofluorescence was carried out as per (Zhang and Levin, 2009), using Invitrogen tyramide kit T12 in Alexa488 (T20922) for amplification. Whole embryos were imaged using Zen software (Zeiss Ltd) to produce tile z-stacks which were then combined to produce a maximum intensity projection. Localization of VANGL2 within the cell was then analyzed using Axiovision software (Zeiss Ltd) to measure the angle between the localization of the VANGL2, the primitive streak and the center of the cell. Statistical analysis was carried out using a Kurtosis distribution and chi squared analysis.

### Wholemount RNA In Situ Hybridization

Mouse and chicken embryos were rehydrated through a methanol gradient and in situ hybridization carried out for chicken *SHH* (Roelink *et al*., 1995), *PITX2* (ChEST ChEST76c15;(Boardman *et al*., 2002), *LEFTY1*, and *NODAL* (kind gifts, IR Paton, the Roslin Institute, Edinburgh, UK; antisense probe- Not1, T3) *FOXJ1* (Cruz *et al*., 2010), *Lefty1/2* probe was a gift from D Norris (MRC Harwell, UK). following the protocol previously described by (Nieto *et al*., 1996).

### Real Time PCR

The primitive streak and some surrounding tissue was dissected from stage 3–4HH embryos and placed immediately into TRIreagent (Sigma) in a FastPrep tubes for dissociation. RNA was isolated and reverse transcription performed using High Capacity cDNA Reverse Transcription Kit (Applied Biosystems). Real-time PCR was performed using Brilliant III Ultrafast SYBR green qPCR master mix (Agilent Technologies) and analysis performed using the Stratagene MX3000 and MxPro software. Expression of *PTCH1* in *wildtype* tissue was normalized to Lamin B Receptor (LBR) expression with genotypes normalized to *wildtype*. This was analyzed using a student's *t*-test and found not to be significant (*P* = 0.84). Expression of *FOXJ1* was normalized to LBR, expression in 20HH hindbrain tissue was 1, in comparison expression in whole embryos at 4HH was 0.6. This was analyzed using a student's *t*-test and found to be significant (*P* = 0.000245).

### Genotyping

Unless stated, all embryos used in comparisons were dissected as family groups and genotyped after analysis. After immunofluorescence or RNA in situ hybridization procedures, embryos were lyzed in 10-mM Tris (pH8), 10-mM EDTA (pH8) 1% SDS, 100-mM NaCl and 20 mg/ml proteinase K at 55°C overnight before DNA extraction using Manual Phase Lock Gel Tubes (5 Prime) for phenol/chloroform extraction. Sequencing primers used were TCATTTCATTAGCTCTGCCG and CCATCAAACCAACAGCTCAG. Mouse embryos were genotyped by PCR using primers-TGCCATGCAGGGATCATAGC;GAGCACACTGGAGGAAAGC; GAGACTCTGGCTACTCATCC; CCTTCAGCAAGAGCTGGGGAC.
