# Supplementary material for: The chicken left right organizer has nonmotile cilia which are lost in a stage-dependent manner in the talpid3 ciliopathy
Source: Genesis. 2014 Apr 15;52(6):600–13. doi: 10.1002/dvg.22775 (PMC4314677; doi:10.1002/dvg.22775)
Supplement: Supplementary file 1 [file dvg0052-0600-sd1.docx]

| **SHH** |  |  |  |  |
| --- | --- | --- | --- | --- |
| **Summary data-** |  |  |  |  |
| **Stage** | **Expression** | **Wildtype** | **Talpid3** | **Unknown Genotype** |
| 3-4HH | Bilateral symmetrical at node | 16 | 2 | 2 |
|  | Left node | 6 | 1 | 1 |
|  | None | 1 | 0 | 0 |
| 5-7HH | Bilateral symmetrical at node | 2 | 0 | 0 |
|  | Left node | 13 | 3 | 3 |
|  | None | 0 | 0 | 0 |
| 8-11HH | Bilateral symmetrical at node | 0 | 0 | 0 |
|  | Left node | 0 | 0 | 0 |
|  | None | 0 | 0 | 0 |
|  | Notochord | 15 | 1 | 6 |

| **LEFTY** |  |  |  |  |
| --- | --- | --- | --- | --- |
| **Summary data-** |  |  |  |  |
| **Stage** | **Expression** | **Wildtype** | **Talpid3** | **Unknown Genotype** |
| 03-4HH | None | 5 | 1 | 0 |
|  | Equal at node | 4 | 0 | 0 |
|  | Left in node | 3 | 0 | 0 |
| 5HH | None | 4 | 0 | 2 |
|  | Right of notochord | 1 | 0 | 0 |
|  | Right notochord, restricted left mesenchyme | 1 | 0 | 0 |
| 6HH | None | 4 | 0 | 0 |
|  | Equal at node | 1 | 0 | 2 |
|  | Rostral notochord | 1 | 0 | 0 |
| 7-9HH | None | 6 | 3 | 4 |
|  | Rostral | 8 | 2 | 3 |
|  | Left notochord | 5 | 0 | 2 |
|  | Right notochord | 0 | 0 | 1 |
|  | Throughout notocord | 2 | 3 | 6 |
|  | Left lateral plate mesoderm | 1 | 1 | 2 |
| 10HH | None | 3 | 1 | 0 |
|  | Nodal | 0 | 0 | 1 |
|  | Left lateral plate mesoderm | 1 | 0 | 1 |
|  | Bilateral in lateral plate mesoderm | 1 | 0 | 1 |
|  | Notocord |  |  | 1 |
